# Zika Virus Infection of Pregnant *Ifnar1*^−/−^ Mice Triggers Strain-Specific Differences in Fetal Outcomes

**DOI:** 10.1128/JVI.00818-21

**Published:** 2021-10-13

**Authors:** Ellie K. Bohm, Jennifer T. Vangorder-Braid, Anna S. Jaeger, Ryan V. Moriarty, John J. Baczenas, Natalie C. Bennett, Shelby L. O’Connor, Michael K. Fritsch, Nicole A. Fuhler, Kevin K. Noguchi, Matthew T. Aliota

**Affiliations:** a Department of Veterinary and Biomedical Sciences, University of Minnesota, Twin Cities, St. Paul, Minnesota, USA; b Department of Pathology and Laboratory Medicine, University of Wisconsin-Madison, Madison, Wisconsin, USA; c Wisconsin National Primate Research Center, University of Wisconsin-Madison, Madison, Wisconsin, USA; d Department of Psychiatry, Washington University in St. Louis, St. Louis, Missouri, USA; University of North Carolina at Chapel Hill

**Keywords:** congenital Zika syndrome, *Flaviviridae*, Zika virus, flavivirus, pregnancy

## Abstract

Zika virus (ZIKV) is a flavivirus that causes a constellation of adverse fetal outcomes collectively termed congenital Zika syndrome (CZS). However, not all pregnancies exposed to ZIKV result in an infant with apparent defects. During the 2015 to 2016 American outbreak of ZIKV, CZS rates varied by geographic location. The underlying mechanisms responsible for this heterogeneity in outcomes have not been well defined. Therefore, we sought to characterize and compare the pathogenic potential of multiple Asian-/American-lineage ZIKV strains in an established *Ifnar1^−/−^* pregnant mouse model. Here, we show significant differences in the rate of fetal demise following maternal inoculation with ZIKV strains from Puerto Rico, Panama, Mexico, Brazil, and Cambodia. Rates of fetal demise broadly correlated with maternal viremia but were independent of fetus and placenta virus titer, indicating that additional underlying factors contribute to fetal outcome. Our results, in concert with those from other studies, suggest that subtle differences in ZIKV strains may have important phenotypic impacts. With ZIKV now endemic in the Americas, greater emphasis needs to be placed on elucidating and understanding the underlying mechanisms that contribute to fetal outcome.

**IMPORTANCE** Zika virus (ZIKV) transmission has been reported in 87 countries and territories around the globe. ZIKV infection during pregnancy is associated with adverse fetal outcomes, including birth defects, microcephaly, neurological complications, and even spontaneous abortion. Rates of adverse fetal outcomes vary between regions, and not every pregnancy exposed to ZIKV results in birth defects. Not much is known about how or if the infecting ZIKV strain is linked to fetal outcomes. Our research provides evidence of phenotypic heterogeneity between Asian-/American-lineage ZIKV strains and provides insight into the underlying causes of adverse fetal outcomes. Understanding ZIKV strain-dependent pathogenic potential during pregnancy and elucidating underlying causes of diverse clinical sequelae observed during human infections is critical to understanding ZIKV on a global scale.

## INTRODUCTION

Zika virus (ZIKV) exposure during pregnancy can cause a constellation of adverse fetal outcomes, collectively termed congenital Zika syndrome (CZS). However, a substantial proportion of pregnancies with *in utero* ZIKV exposure result in babies without apparent defects. Only an estimated 5 to 15% of exposed infants have ZIKV-related birth defects (1–3). Importantly, infants who are born apparently healthy can manifest developmental and neurocognitive deficits months to years after birth (4–7), even if maternal exposure resulted in asymptomatic infection (8). Furthermore, there was an unequal distribution of ZIKV cases and severe outcomes in all areas where ZIKV emerged in the Americas, demonstrating that risk of CZS varied over time and with geographic location (reviewed in reference 9). For example, the rate of microcephaly differed between French Polynesia (1%) (9), the U.S. Territories and Freely Associated States (5 to 6%) (10), and the Dominican Republic (11%) (7). Within Brazil, the rate of microcephaly varied between São Paulo (0%) (11), Pernambuco (2.9%) (12), Rio de Janeiro (3.5%) (13), Southeast Brazil (1.5%), and Northeast Brazil (13%) (14). However, it should be noted that accurate diagnosis of microcephaly requires multiple measures after birth and the use of inconsistent definitions of cases and complications can bias reporting (15). For example, initial microcephaly rates were overestimated in Brazil before INTERGROWTH-21st reference-based standards were implemented (16).

Microcephaly is not the only adverse birth outcome that results from gestational ZIKV infection (17), and these rates varied as well. The U.S. Territories and Freely Associated States reported birth defects in 14% of ZIKV-exposed pregnancies (10). Pernambuco, Brazil reported adverse outcomes in 20% of exposed pregnancies (12), whereas São Paulo reported a 28% rate of adverse neurological outcomes (11). Strikingly, in Rio de Janeiro, 42% of infants born to ZIKV-exposed pregnancies had adverse outcomes; however, this study used a broader definition for ZIKV-associated outcomes (13). Because current diagnostic testing remains suboptimal and inconsistent for the detection of congenital ZIKV infection (18), the relative risk of CZS in infants from ZIKV-exposed pregnancies remains unknown, and it remains unknown whether the risk is equal in different geographic areas. Was the unequal distribution in CZS incidence over time and region stochastic or were there other factors that influenced these regional differences? A provocative explanation for the appearance of CZS in the Americas is that contemporary ZIKV strains evolved from strains that cause fetal lethality to those that cause birth defects and this may have facilitated recognition of ZIKV’s ability to harm the developing fetus (19). Whether ongoing virus evolution during geographic spread in the Americas gave rise to phenotypic variants that differ in their capacity to harm the developing fetus remains an open question.

Large case-control studies of pregnant women may prove useful for determining whether the infecting ZIKV genotype affects overall pathogenesis during pregnancy. However, these types of studies are observational and are complicated by participant heterogeneity, including history of infection with other flaviviruses, and the precise time, dose, and genetic makeup of the infecting virus. We therefore aimed to better understand heterogeneity in ZIKV-associated pregnancy outcomes by investigating whether there are Asian-/American-lineage strain-specific phenotypic differences by using mice lacking type I interferon signaling (*Ifnar1^−/−^*). Although there are limitations regarding the translational relevance of this model, transplacental ZIKV infection and fetal damage have been demonstrated (20–23), and it has been used to compare maternal infection parameters, placental pathology, fetal infection, and outcomes between ZIKV strains and the closely related Spondweni virus (20–22). Congenital ZIKV studies in pregnant mouse models have used a variety of virus strains, as well as timing, route, and dose of inoculation (22–25). This heterogeneity in design has made it difficult to compare results across mouse studies because both inoculation dose and time of ZIKV exposure during pregnancy play a role in determining fetal outcomes (26). Therefore, we assessed fetal outcomes following infection by a panel of five geographically distinct, low-passage Asian-/American-lineage ZIKV strains at embryonic day 7.5 (E7.5). Here, we found that all ZIKV strains infected the placenta but varied in their capacity to cause overt fetal harm, suggesting there is phenotypic heterogeneity in pregnancy outcomes that is dependent on the infecting ZIKV genotype.

## RESULTS

### ZIKV replication kinetics in maternal serum are broadly similar among Asian-/American-lineage strains.

To perform a comprehensive phenotypic characterization of ZIKV infection during pregnancy, we assembled a set of five recently isolated, low-passage ZIKV strains based on their geographic distribution in the Americas and minimal passage history. Our ZIKV panel included four epidemic strains from the American-sublineage (Puerto Rico-2015, PR; Panama-2015, PAN; Mexico-2016, MEX; and Brazil-2015, BRA) and a nonepidemic strain from the Asian-lineage (Cambodia-2010, CAM). ZIKV-PR, ZIKV-PAN, ZIKV-MEX, and ZIKV-BRA share >99.5% genome-wide nucleotide identity, and ZIKV-CAM shares over 98% genome-wide nucleotide identity, resulting in only 4 to 18 amino acid differences between strains (Tables 1 and 2).

**TABLE 1:** Total number of amino acid differences between strains and percent difference in amino acid identity

Strain	No. amino acid differences (%)			
	CAM	BRA	MEX	PAN
PR	17 (0.50)	4 (0.12)	9 (0.26)	7 (0.20)
PAN	16 (0.47)	5 (0.15)	8 (0.23)	
MEX	18 (0.53)	7 (0.20)		
BRA	15 (0.44)			

**TABLE 2:** Differences in amino acid sequences across Asian-/American-Lineage ZIKV strains^*a*^*^,^*^*b*^

PR	PAN	MEX	BRA	CAM	Protein^*c*^	Codon
**T**	I	I	I	I	C	80
A	A	A	A	**T**	C	106
D	**E**	D	D	D	C	107
A	A	A	A	**V**	prM	1
S	S	S	S	**N**	prM	8
N	N	N	N	**S**	prM	17
L	L	L	L	**M**	prM	29
**L**	V	V	V	V	E	330
M	M	M	M	**V**	E	473
G	G	**A**	G	G	NS1	100
V	V	V	V	**A**	NS1	188
R	**W**	R	R	R	NS1	324
K	K	**E**	K	K	NS1	326
M	M	M	**V**	M	NS1	349
L	L	L	L	**P**	NS2a	128
T	T	T	T	**X**	NS2b	105
V	V	**I**	V	V	NS3	40
**F**	S	S	**F**	S	NS3	356
M	M	**L**	M	M	NS3	572
H	H	H	H	**Y**	NS3	584
**V**	A	A	A	A	NS5	91
V	V	V	V	**M**	NS5	114
I	I	**T**	I	I	NS5	526
T	**A**	T	T	T	NS5	833
V	V	V	V	**M**	NS5	872
M	M	M	M	**V**	NS5	883

a PR (PRVABC59; GenBank AMC13911.1); PAN (259249; GenBank ANB66183); MEX (R116265; GenBank AOG18296.1); CAM (FSS13025; GenBank AFD30972); BRA (Paraiba_01; GenBank ANH10698.1).

b Boldface text indicates a variant amino acid.

c C, capsid; prM, precursor membrane; E, envelope; NS, nonstructural.

**FIG 1:**
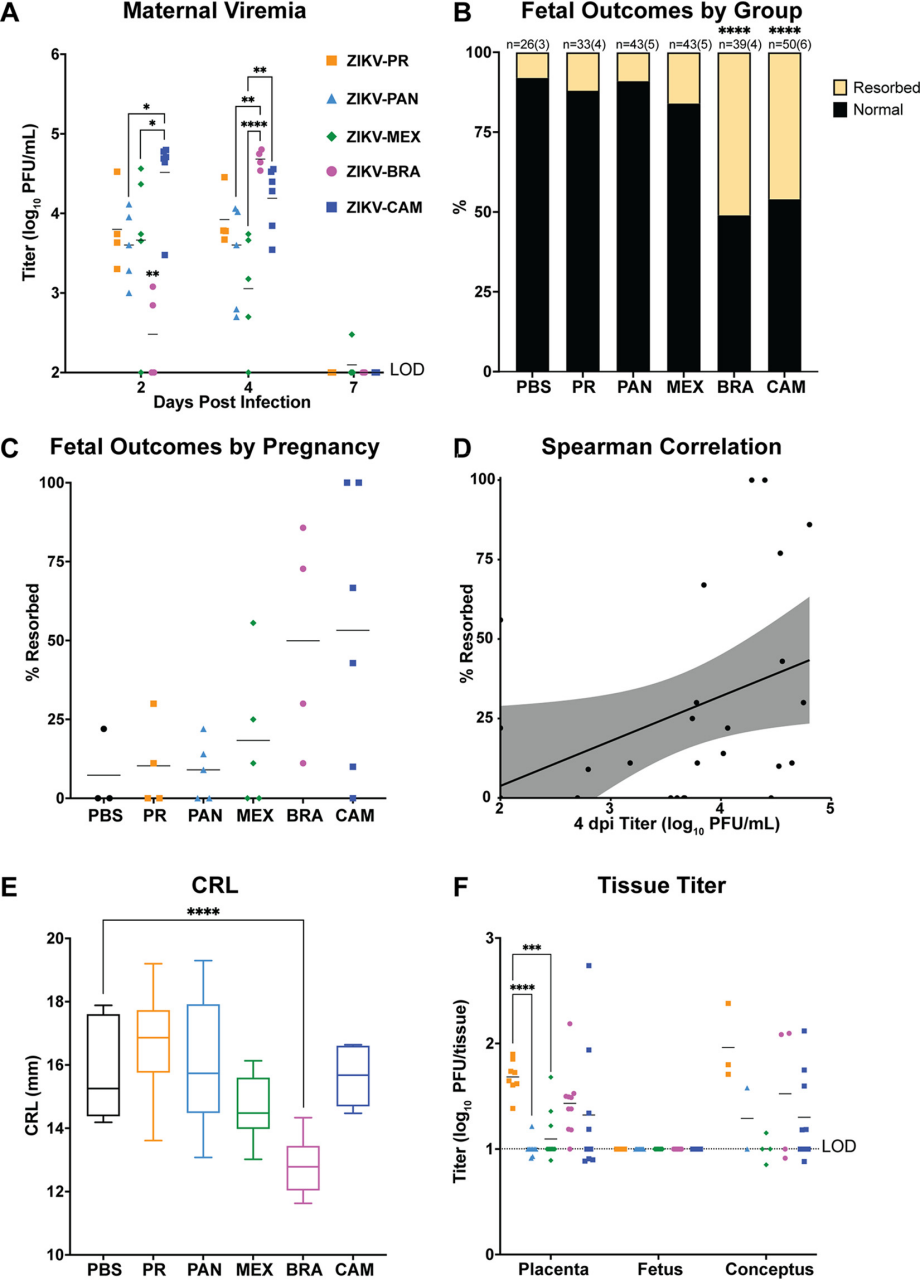
ZIKV strains are phenotypically heterogeneous. (A) Time-mated *Ifnar1*^−/−^ dams were inoculated with 10^3^ PFU of ZIKV on E7.5 and maternal infection was assessed by plaque assay on 2, 4, and 7 days postinoculation (dpi), and significance was determined by one-way ANOVA. (B) Rate of normal (black) versus resorbed (yellow) fetuses at E14.5 after maternal infection at E7.5. Data presented are *n* = number of individual fetuses (from 3 to 6 litters per treatment group). Significance was determined by Fisher’s exact test. (C) Pregnancy outcomes of individual animals in each treatment group. Data are presented as percent of fetuses resorbed in each pregnancy. (D) Spearman correlation of maternal serum titer at 4 dpi versus resorption rate (*r* = 0.5200, *P* value = 0.0054). (E) Crown-to-rump length (CRL) measurements in mm of morphologically normal fetuses at E14.5 using ImageJ software. Significance was determined by one-way ANOVA. (F) Tissue titer was measured by plaque assay for individual homogenized placentas, fetuses, and concepti (when the fetus and placenta were indistinguishable due to severe resorption). Symbols represent individual placenta, fetus, or conceptus from 4 to 6 independent experiments for each treatment group. Bars represent the mean viral titer of each treatment group and significance was determined by one-way ANOVA. Significance annotations for all figures: ****, *P* ≤ 0.0001; ***, *P* ≤ 0.001; **, *P* ≤ 0.01; *, *P* ≤ 0.05.

To characterize the range of pathogenic outcomes and assess the effect of ZIKV strain on *in utero* exposure, we utilized a well-established murine pregnancy model for ZIKV (20, 21). *Ifnar1*^−/−^ dams were time-mated with wild-type (WT) males to produce fetuses and a maternal-fetal interface (MFI) with intact interferon (IFN) signaling. Pregnant *Ifnar1*^−/−^ dams were inoculated with 1 × 10^3^ PFU of ZIKV-PR, ZIKV-PAN, ZIKV-MEX, ZIKV-BRA, or ZIKV-CAM via subcutaneous footpad inoculation at embryonic day 7.5 (E7.5), corresponding to the mid-to-late first trimester in humans (27). Based on results from our past studies (20, 21), we chose this dose to minimize the potential confounding impacts of maternal illness on fetal outcomes. Maternal serum samples were collected at 2, 4, and 7 days postinoculation (dpi) to confirm infection and examine viremia kinetics (Fig. 1A). All dams were productively infected with detectable viremia by 4 dpi for all groups. ZIKV-CAM replicated to significantly higher titers at 2 dpi compared to ZIKV-PAN and ZIKV-MEX (one-way ANOVA with Tukey’s multiple comparisons, *P = *0.0199 and *P = *0.0392, respectively), whereas maternal viremia was significantly lower in ZIKV-BRA-inoculated dams compared to all other treatment groups at this time point. By 4 dpi, ZIKV-BRA had replicated to significantly higher titers compared to ZIKV-PAN and ZIKV-MEX (*P = *0.0026 and *P* < 0.0001, respectively). Maternal viremia in the ZIKV-CAM group also was significantly higher compared to the ZIKV-MEX group at 4 dpi (*P = *0.0024). Overall, maternal viremia reached similar levels before being cleared to the limit of detection by 7 dpi in all groups. Due to the impact of COVID-19, ZIKV-PAN maternal serum samples at 7 dpi were not collected. Dams were monitored daily for clinical signs until the time of necropsy at E14.5 (7 dpi) and no overt clinical signs were observed in any virus- or PBS-inoculated dams.

### Adverse fetal outcomes are dependent on the infecting ZIKV strain.

Next, to assess fetal outcomes, dams were necropsied on E14.5. Gross examination of each conceptus revealed overt differences among fetuses within pregnancies and with uninfected counterparts. Fetuses appeared as either morphologically normal or undergoing embryo resorption, as defined in reference 20. At the time of necropsy, we observed high rates of resorption from ZIKV-BRA- and ZIKV-CAM-infected pregnancies (ZIKV-BRA: 51% and ZIKV-CAM: 46%), which were significantly higher than the other virus-inoculated groups and PBS-inoculated controls (Fisher’s exact test, *P < *0.0001) (Fig. 1B). In contrast, the proportion of resorbed fetuses for ZIKV-PR-, ZIKV-PAN-, and ZIKV-MEX-infected pregnancies did not differ significantly from each other or from PBS-inoculated controls (ZIKV-PR: 12%, ZIKV-PAN: 9%, and ZIKV-MEX: 16%, PBS: 8%, *P > *0.1264) (Fig. 1B). The rate of embryo resorption also varied between individual pregnancies within each treatment group (Fig. 1C). Maternal viremia at 4 dpi positively correlated with increased fetal resorption across all virus groups (Spearman, *P = *0.0111) (Fig. 1D), but this trend was not observed within individual virus groups (Spearman, *P > *0.1333). Therefore, our results demonstrate that multiple ZIKV genotypes differ in their propensity to cause fetal harm in this experimental model and additional factors, beyond maternal infection, may contribute to fetal outcome.

### Fetal growth restriction is only evident following ZIKV-BRA infection.

To further characterize pathogenic outcomes during pregnancy, we measured crown-to-rump length (CRL) to assess overall fetal growth (20, 22). Only fetuses that appeared morphologically normal were included for measurement of CRL to provide evidence for intrauterine growth restriction (IUGR). There was a statistically significant reduction in CRL in ZIKV-BRA fetuses compared to fetuses from PBS-inoculated dams (one-way ANOVA with Tukey’s multiple comparisons, *P < *0.0001) (Fig. 1E). In contrast, mean CRL did not differ significantly between fetuses from ZIKV-MEX-, ZIKV-CAM-, ZIKV-PAN-, ZIKV-PR-, and PBS-inoculated dams (*P > *0.1561). The lack of apparent IUGR for the other ZIKV strains is contrary to other studies using Asian-lineage ZIKVs (French Polynesia and Cambodia), in which fetuses developed severe IUGR (22, 23). However, the discrepancy in outcomes may be the result of differences in timing of challenge and necropsy, dose and/or route of inoculation, dam age, litter size, or metrics for defining grossly normal fetuses compared to those undergoing resorption at a later embryonic age.

### No evidence for vertical transmission in any virus treatment groups.

Next, to determine the potential of each ZIKV strain to be vertically transmitted, a subset of placentas and fetuses were collected for plaque assay at the time of necropsy from each litter in all treatment groups. No infectious virus was detected by plaque assay in any fetus sample from any treatment group (Fig. 1F) and the absence of ZIKV fetal infection was confirmed by RNA *in situ* hybridization (ISH). In contrast, virus was detected in placentas from all virus-inoculated groups at the time of necropsy at E14.5 (7 dpi). ZIKV-PR placenta titers were significantly higher than ZIKV-PAN and ZIKV-MEX titers (one-way ANOVA with Tukey’s multiple comparisons, *P* < 0.0001 and *P = *0.0006, respectively), but only modestly higher than ZIKV-CAM and ZIKV-BRA titers (*P = *0.5591 and *P = *0.5693, respectively) (Fig. 1F). In ZIKV-PR, ZIKV-PAN, ZIKV-MEX, and ZIKV-BRA groups, placenta titer was not a predictor of partner fetus outcome (one-way ANOVA, *P > *0.0970). Although limited by the number of data points, ZIKV-CAM placentas with resorbed fetuses had significantly higher titers than those from normal fetuses (*P = *0.0182). Therefore, we hypothesized that additional factors, outside of fetus and placenta virus levels, contribute to poor fetal outcomes.

### Severe placental histopathological changes were consistently detected in ZIKV-CAM-infected mice.

**FIG 2:**
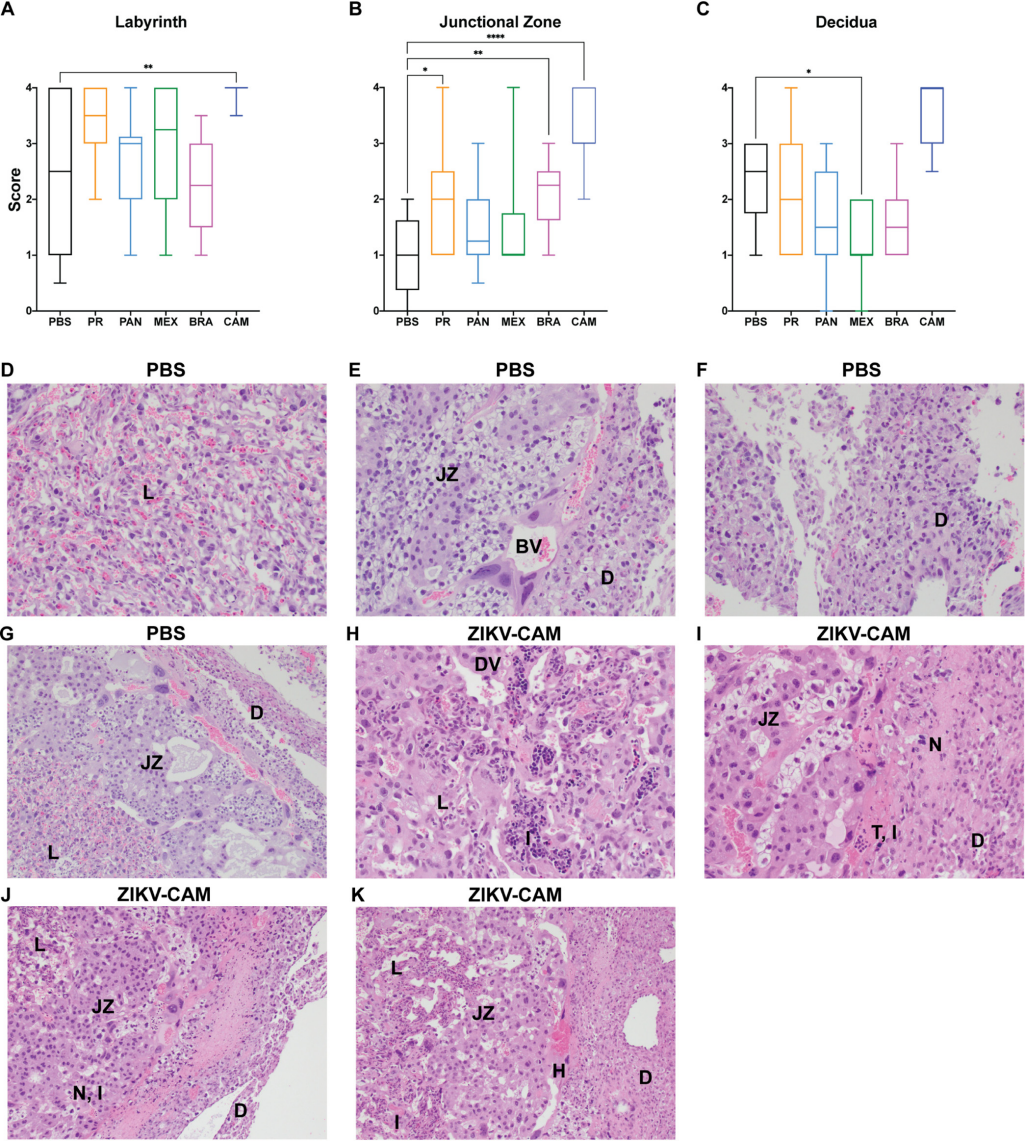
Placenta histopathology is dependent on infecting virus strain. (A to C) The degree of placenta pathology was rated on a scale of 0 to 4, where 0 represents normal histologic features and 4 represents the most severe features observed. Each zone of the placenta was scored individually for general overall pathology, amount of inflammation, and amount of vascular injury. Only “general” scores are shown because they are representative of “inflammation” and “vascular injury” categories and do not differ significantly from “general.” Box and whiskers represent the minimum-to-maximum of all data points around the median. Data are representative of 3 to 6 animals per treatment group. ****, *P* ≤ 0.0001; **, *P* ≤ 0.01; *, *P* ≤ 0.05 (Kruskal-Wallis ANOVA). (D to G) Normal histologic features of each placental zone from PBS-inoculated dams. Labyrinth (L); junctional zone (JZ); decidua (D); normal blood vessel (BV) between the junctional zone and decidua. (H to K) Severe histopathologic injury patterns from placentas from ZIKV-CAM-inoculated dams. (H) Dilated vasculature (DV) and inflammation (I) in the labyrinth. (I) Thrombus (T) and inflammation at the junction of the junctional zone and decidua, and necrosis (N) in the decidua. (J) Necrosis and inflammation at the junction of the junctional zone and decidua, and necrosis of the decidua. (K) Dilated vascular space and inflammation in the labyrinth, hemorrhage (H) in the junctional zone, and necrosis in the decidua.

To better characterize the impact of *in utero* infection of different ZIKV strains, placental tissues were examined microscopically (Fig. 2). In phosphate-buffered saline (PBS)-inoculated dams, we observed normal decidua, junctional zone, and labyrinth, with normal maternal and fetal blood spaces (Fig. 2A to G). In contrast, ZIKV-inoculated dams displayed varying degrees of placental pathology (Fig. 2A to C), similar to what we have reported previously, with the most severe effects predominantly observed in the labyrinth zone, including necrosis, calcifications, thrombi, inflammation, and apoptosis (20, 21). Interestingly, the overall severity observed within virus groups was relatively subtle compared to our previous studies (20, 21), which may account for the higher background scores noted in the PBS control group (Fig. 2A to C). There also were clear strain-specific differences in the amount of placental pathology observed, with ZIKV-CAM displaying the most severe histologic phenotype (Fig. 2H to K). Similar to placenta titer, pathology severity score was not a predictor of adverse fetal outcome for any treatment group.

### Interferon-stimulated gene transcript abundance is elevated in placentas after ZIKV infection.

**FIG 3:**
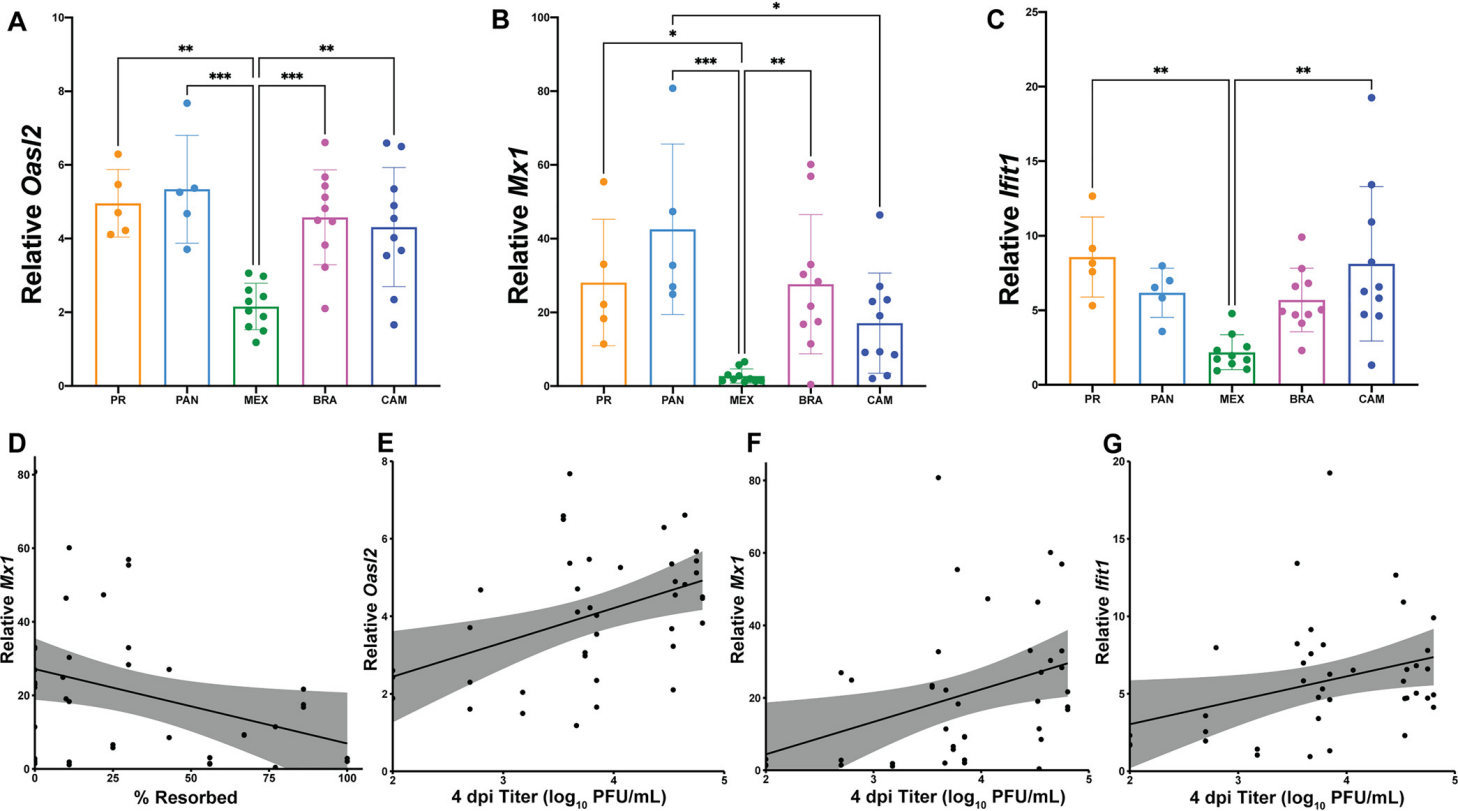
Interferon-stimulated gene expression is elevated in placentas from ZIKV-infected dams. (A to C) RNA was extracted from placentas harvested on E14.5 and expression of the ISGs *Oasl2* (A), *Mx1* (B), and *Ifit1* (C) were analyzed by qPCR. Levels were normalized to *Gapdh* and then dd*C_T_* was calculated relative to placentas harvested from PBS-inoculated dams. One to four placentas from two litters of PBS-inoculated and four to five litters of each ZIKV treatment group were analyzed. Means with standard deviation are shown. ***, *P* ≤ 0.001; **, *P* ≤ 0.01; *, *P* ≤ 0.05 (one-way ANOVA). (D to G) Spearman correlations with shaded 95% confidence interval are shown for % resorbed versus relative *Mx1* expression (r = −0.6593; *P* value = 0.0169) (D), and 4 dpi maternal titer versus relative expression of *Oasl2* (*r* = 0.3752; *P* value = 0.0171) (E), *Mx1* (*r* = 0.4111; *P* value = 0.0084) (F), and *Ifit1* (*r* = 0.3137; *P* value = 0.0487) (G).

Due to the lack of vertical transmission and an association between fetal outcome and placenta infection and pathology, we hypothesized that interferon (IFN) induction in the placenta was responsible for determining fetal outcome. Indeed, it has previously been shown that type I IFN signaling, not the levels of virus, mediated pathology following intravaginal ZIKV infection in *Ifnar1^+/−^* fetuses and placentas (23). Accordingly, we examined the transcriptional changes of the interferon-stimulated genes (ISGs) *Oasl2*, *Mx1*, and *Ifit1* in the placenta as markers of IFN signaling activation to determine if IFN induction (or the lack thereof) may be contributing to fetal demise. Mice on the C57BL/6 background carry nonfunctional alleles of the *Mx1* gene (28), and *Mx1* is not a relevant ISG that restricts ZIKV infection (29). As a result, we are using *Mx1* transcript abundance as an indirect measure of global IFN signaling activity in the placenta, in concert with *Oasl2* and *Ifit1*, to begin to understand if there may be differential activation of innate immune mechanisms between strains. We observed that *Oasl2*, *Mx1*, and *Ifit1* were induced regardless of infecting ZIKV genotype in our model (Fig. 3A to C). Interestingly, ZIKV-MEX placentas had significantly lower *Mx1*, *Oasl2*, and *Ifit1* transcript abundance compared to the other virus groups (one-way ANOVA, *P < *0.0357). Across virus groups, pregnancies with better outcomes (i.e., lower rates of resorption) had higher expression of *Mx1* (Spearman, *P = *0.0169) (Fig. 3D), and ZIKV-CAM placentas with normal fetuses expressed higher levels of *Mx1* than their resorbed counterparts (*P = *0.0464; mean ± standard error of the mean [SEM]: −18.01 ± 7.650; *n* = 10). However, there was no correlation between ISG expression and pregnancy outcome within any virus group (Spearman, *P > *0.0833). Also, ISG expression and placenta histopathology scores showed no clear relationship. Across virus groups, maternal serum titer at 4 dpi positively correlated with increased expression of *Oasl2*, *Mx1*, and *Ifit1* in the placenta (Spearman, *P < *0.0487) (Fig. 3E to G). These data suggest a neutral, or modestly protective, role for the IFN response in our model.

**FIG 4:**
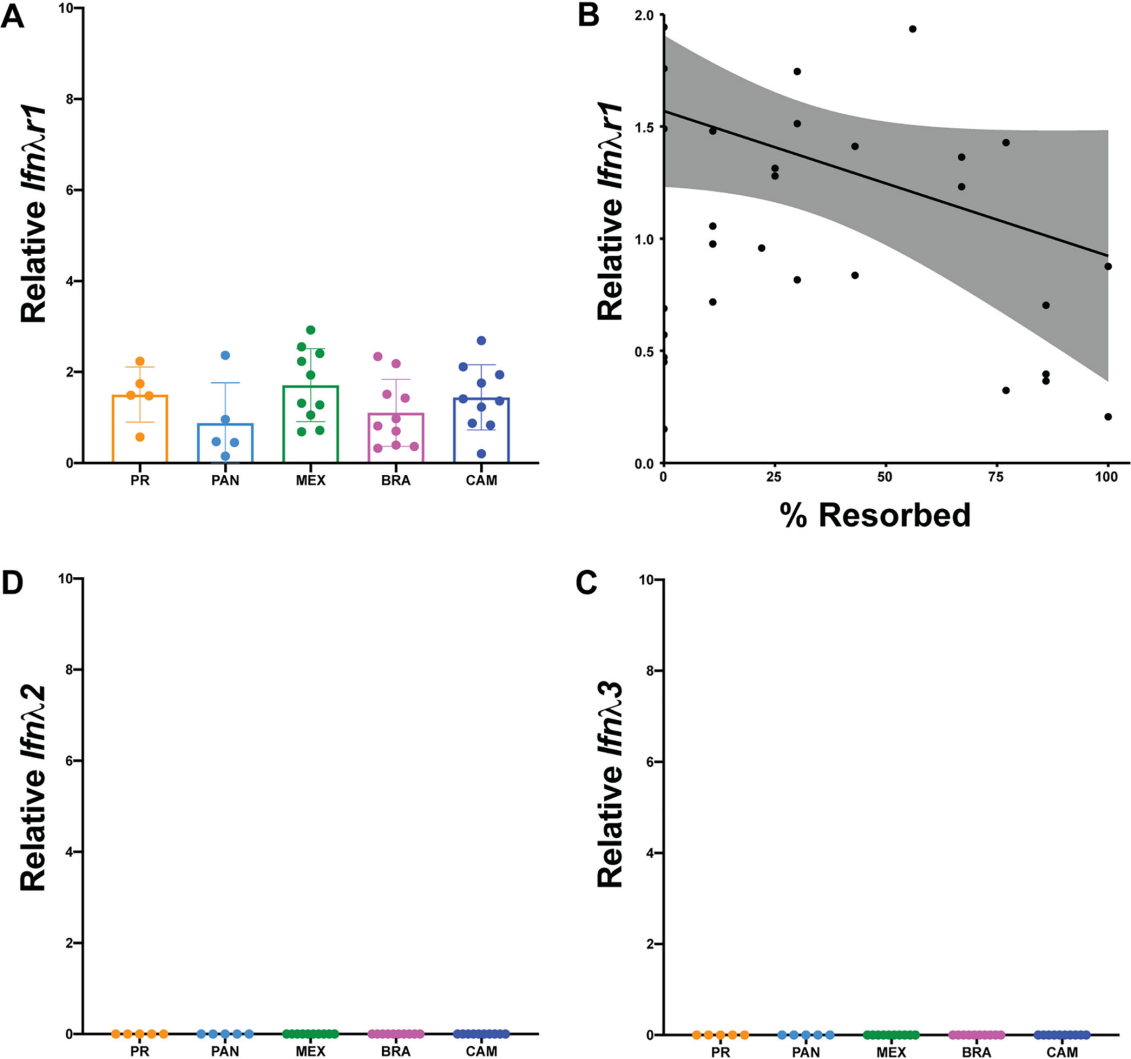
No robust evidence for type III IFN induction. RNA was extracted from placentas harvested on E14.5 and relative expression was analyzed by qPCR. Levels were normalized to *Gapdh* and then dd*C_T_* was calculated relative to placentas harvested from PBS-inoculated dams. (A) *Ifnλr1* expression. (B) Spearman correlation of % resorbed and *Ifnλr1* expression from individual pregnancies (r = −0.6099; *P* value = 0.0302). (C and D) Relative expression of IFNλ2 (C) and IFNλ3 (D).

We then examined if infection outcomes were due to differential expression of IFN-λ or its heterodimeric receptor *Ifnλr1/Il10rβ*. We measured the relative transcript abundance of *Ifnλr1*, *Ifnλ2*, and *Ifnλ3* in the placenta at time of necropsy at E14.5. *Ifnλ1* is a pseudogene and the genomic region of *Ifnλ4* is missing in mice (30) and, therefore, was not measured. Consistent with our ISG data, we observed modest induction of *Ifnλr1* for all ZIKV strains (Fig. 4A). Importantly, pregnancies with lower rates of resorption had higher expression of *Ifnλr1* (Spearman, *P = *0.0302) (Fig. 4B), and ZIKV-CAM placentas with normal fetuses had higher expression of *Ifnλr1* than their resorbed counterparts (*P = *0.0087; mean ± SEM: −1.148 ± 0.333; *n* = 10). In contrast, *Ifnλ2* and *Ifnλ3* were not induced in any placenta sample from ZIKV-infected mice (Fig. 4C and D). This, perhaps, was not surprising, since a previous mouse study showed that type III IFNs played little to no role in placental antiviral defenses before placentation (25). In our model, dams were infected on E7.5 and placental development is not complete until E8.5 to 10.5 in mice (31, 32). Still, it remains unknown whether the mouse placenta constitutively releases type III IFNs in a manner similar to the human placenta, or whether these IFNs are induced systemically or in response to placental infection (33). Here, we did not detect robust evidence for induction of type III IFNs despite detection of infectious virus in the placenta at the time of necropsy at E14.5.

## DISCUSSION

By comparing five ZIKV strains representing the viral genetic diversity in the Americas (34), we provide experimental evidence that there is strain-dependent phenotypic heterogeneity in pregnancy outcomes following *in utero* ZIKV exposure (summarized in Table 3). In our pregnant *Ifnar1^−/−^* mouse model, ZIKV-CAM and ZIKV-BRA caused significantly more embryo resorption than ZIKV-PR, ZIKV-PAN, and ZIKV-MEX at E14.5. Maternal infection with ZIKV-PAN and ZIKV-MEX resulted in low levels of placenta infection with varying degrees of placenta pathology, and overall low rates of embryo resorption. In contrast, ZIKV-PR replicated to high titers in the placenta and this corresponded with severe histopathology, but did not result in fetal demise. No strain resulted in detectable fetal infection, which is different from what we have reported previously with African-lineage ZIKV (20, 21). However, the absence of ZIKV fetal infection does not preclude the possibility that pathology may develop later in pregnancy or even postnatally, similar to what has been observed in humans (35). Importantly, we only assessed pregnancy outcomes at a single necropsy time point (E14.5) and outcomes may differ depending on the gestational age of the fetus (36). It is possible that assessments at a later gestational age, as pathology continues to manifest, may reveal more, or less, striking differences in pregnancy outcomes between ZIKV strains. It is unknown whether adverse pregnancy outcomes in humans require direct infection of the fetus (i.e., vertical transmission) or whether pathophysiology at the maternal-fetal interface (MFI) without vertical transmission is sufficient to cause adverse outcomes. Placental insufficiency is now being recognized as a potential contributor to some of these adverse outcomes (37, 38), and our data suggest that pregnancy loss is not solely driven by fetal infection. However, it is unclear to what extent this mouse model recapitulates ZIKV infection and disease in humans. The structure of the placenta and the organization of the maternal-fetal interface of mice are significantly different than in humans, making it difficult to directly compare the pathological consequences observed between hosts.

**TABLE 3:** Summary of observed ZIKV strain infection outcomes

Strain	% Fetal resorption	IUGR^*a*^	Vertical transmission	Placenta infection	Severe placenta histopathology	IFN^*a*^ activation
PBS (control)	8					
ZIKV PR	12			x		high
ZIKV PAN	9			x		high
ZIKV MEX	16			x		low
ZIKV BRA	51	x		x		high
ZIKV CAM	46			x	x	high

a IUGR, intrauterine growth restriction; IFN, interferon.

One possible explanation for differences in fetal outcomes observed between treatment groups could be due to differences in activation of and/or susceptibility to antiviral signaling at the MFI. It is becoming increasingly apparent that IFN responses can have protective and/or pathogenic effects in pregnancy (reviewed in reference 30). Protection associated with IFN production prevents uncontrolled virus replication, fetal infection, and maternal mortality (39–41); however, overproduction of type I IFNs are known to be an underlying cause of pregnancy complications, including developmental defects similar to those that result from infections with teratogenic pathogens (23, 42, 43). As a result, there likely is a critical balance that must occur between the beneficial antiviral effects of the IFN response to virus infections during pregnancy and the pathological consequences that may result from excessive production of IFNs. We examined the relative levels of the ISGs *Oasl2*, *Mx1*, and *Ifit1* in the placenta because of their known relevance to mouse (23, 44–47) and nonhuman primate (48) models of ZIKV infection, broad-spectrum antiviral functions (49–53), contributions to placental pathology (42, 54), and general involvement in the success of human pregnancies (30, 39, 55). Our data suggest that IFN activation did not contribute to fetal demise and, in some cases, may have played a protective role. Maternal viremia appeared to drive ISG induction in the placenta, which may not be surprising given that the mouse labyrinth is perfused with maternal blood (56). Higher maternal viremia also positively correlated with increased resorption rate across virus groups. Therefore, maternal viremia may also contribute to an increased risk of adverse fetal outcomes, alone or in combination with IFN-dependent causes, direct pathogenic effects of the virus, or as a bystander effect associated with immune responses unrelated to IFN induction. Similar to resorption rate, we only assessed ISG expression at a single time point, at time of necropsy (E14.5, 7 dpi), and expression profiles may differ depending on the timing of collection. More studies are needed to better understand antiviral signaling at the MFI and the mechanisms these virus strains exploit to harm the feto-placental unit.

Differences observed in fetal outcomes and histopathology across ZIKV strains may also be due to virus genetic determinants of virulence and pathogenesis during congenital infection. Because contemporary ZIKV isolates are so closely related, they are oftentimes used interchangeably in laboratory research. But even though there is high genetic similarity between ZIKV strains, these subtle genotypic differences likely result in small, but biologically important, phenotypic differences between strains. For example, evidence suggests that ZIKV virulence can be governed both by viral nucleotide sequence and/or amino acid sequence (44, 57–61). Indeed, other groups have reported an amino acid change (E-V330L) common to laboratory-passaged ZIKV-PR stocks that might attenuate viral pathogenicity in nonpregnant adult mice (57, 61). Our ZIKV-PR stock primarily contains the L330 variant (Table 4) and produced robust placenta infection but only low rates of fetal resorption at E14.5. We cannot determine whether using a ZIKV-PR stock with majority V330 variant would have affected the results we report here. Similarly, a ZIKV-BRA stock with NS2A-A117V may enhance viral pathogenicity in nonpregnant adult mice (44). Our ZIKV-BRA stock is majority A117 (Table 2) and resulted in significant rates of fetal demise. Finally, a recent study tested a panel of amino acid substitutions, including prM-A123V, NS1-G894A, NS3-M2074L, and NS3-H2086Y in an otherwise isogenic background in a ZIKV-PR infectious clone, and found that the V123, A894, and L2074 variants resulted in higher rates of fetal demise (59). Our ZIKV-CAM stock is majority V123 but our ZIKV-BRA stock is majority A123 (Table 2)—and both had high rates of fetal resorption. In contrast, our ZIKV-MEX stock is the only stock that is majority A894 and L2074 (Table 2) and this strain had low rates of fetal resorption. In sum, the impact of a single amino acid substitution may vary in the different strains chosen for analyses (20, 62). Because our experiment was not designed to directly link virus genotype to phenotype using reverse genetics, we cannot rigorously determine which single nucleotide polymorphisms, singly or in combination, may promote the strain phenotypes we observed. Future reverse genetic studies will be needed to link viral genotypes to fetal pathogenicity in this model.

**TABLE 4:** Nucleotide variants in challenge stocks relative to the GenBank reference sequence. Only variants found in >5% of sequences are shown

Isolate	Mutation	Nucleotide position	Frequency (%)	Amino acid change	Protein	Codon position
Challenge stock: Zika virus/H.sapiens-tc/PUR/2015/PRVABC59-3329. Reference: KU501215.1	G → T	1964	92.06	V → L	E	330
	T → G	2780	5.36	W → G	NS1	98
	T → C	3147	12.27	M → T	NS1	220
	C → T	5679	54.31	S → F	NS3	356
	C → T	7915	10.56	None (G)	NS5	83
Challenge stock: Zika virus/H.sapiens/PAN/2015/PA 259249. Reference: KX156775	→ A	67/68	6.34	Frameshift	C	4
	→ A	275/276	7.56	Frameshift	C	73
	C → T	440	7.87	None (R)	prM	6
	T → C	2611	7.42	F → S	NS1	58
Challenge stock: Zika virus/H.sapiens/MEX/2016/R116265. Reference: KX766029	→ A	321/322	5.06	Frameshift	C	73
	C → T	3138	12.47	None (I)	NS1	218
	T → C	8118	5.67	None (S)	NS5	152
	C → R	8348	12.8	T → I	NS5	229
	A → T	9753	13.82	K → N	NS5	697
	G → T	10681	6.67		3′ UTR	
	G → T	10687	10		3′ UTR	
Challenge stock: Zika virus/H.sapiens/Brazil/2015/Paraiba_01. Reference: KX280026.1	A → G	693	18.75	T → A	prM	74
	T → C	798	18.82	S → P	M	16
	C → T	970	40.91	A → V	M	73
	C → T	4184	43.32	None (N)	NS2A	212
	G → T	4994	9.57	None (A)	NS3	127
	C → T	5680	54.21	S → F	NS3	356
	T → C	5693	48	None (V)	NS3	360
	A → G	6373	18.76	K → R	NS3	587
	C → A	7943	45.68	None (A)	NS5	92
	G → T	8281	6.99	G → V	NS5	205
Challenge stock: Zika virus/H.sapiens-tc/CAM/2010/FSS13025-7376. Reference: JN860885	No changes					

Our findings highlight that phenotypic heterogeneity exists between closely related ZIKV strains that are commonly used for pathogenesis studies. To more rigorously assess the relative capacity of Asian-/American-lineage ZIKVs to cause adverse fetal outcome, future studies should carefully consider the specific characteristics of the virus strains being used and consider them in the specific context of the questions being asked. One important limitation to our study is that it remains unclear whether the same phenotypes would be recapitulated during human infection. Further, we do not argue that the phenotypic differences we observe between strains indicate diminished risk of adverse outcomes following infection during pregnancy with a certain ZIKV genotype (63). On the contrary, the presence of infectious ZIKV in the placenta for all strains tested is concerning and suggests that all ZIKV strains have the capacity to harm the developing fetus depending on the specific pathophysiological context of infection at the MFI. Here, our results provide a comparative framework to further investigate underlying factors that determine fetal outcome during *in utero* ZIKV exposure.

## MATERIALS AND METHODS

### Ethical approval.

This study was approved by the University of Minnesota, Twin Cities Institutional Animal Care and Use Committee (Animal Care and Use protocol number 1804-35828).

### Cells and viruses.

African green monkey kidney cells (Vero cells; ATCC CCL-81) were maintained in Dulbecco’s modified Eagle medium (DMEM) supplemented with 10% fetal bovine serum (FBS; Corning, Manassas, VA), 1× Antibiotic Antimycotic solution (Corning, Manassas, VA) and incubated at 37°C in 5% CO_2_. Aedes albopictus mosquito cells (C6/36; ATCC CRL-1660) were maintained in DMEM supplemented with 10% fetal bovine serum (FBS; HyClone, Logan, UT), 2 mM l-glutamine, 1.5 g/liter sodium bicarbonate, 1× Antibiotic Antimycotic solution, and incubated at 28°C in 5% CO_2_. The cell lines were obtained from the American Type Culture Collection, were not further authenticated, and were not specifically tested for mycoplasma.

ZIKV strain PRVABC59 (ZIKV-PR; GenBank KU501215) was originally isolated from a traveler to Puerto Rico in 2015 with three rounds of amplification on Vero cells. ZIKV strain R116265 (ZIKV-MEX; GenBank KX766029) was originally isolated from a 73-year-old-male traveling in Mexico in 2016 with a single round of amplification on Vero cells (CDC, Ft. Collins, CO). ZIKV strain 259249 (ZIKV-PAN; GenBank KX156775) was originally isolated from a human serum sample from Panama in 2015 with two rounds of amplification on Vero cells, followed by one round of amplification on C6/36 mosquito cells. ZIKV strain FSS13025 (ZIKV-CAM; GenBank JN860885) was originally isolated from a child in Cambodia with three rounds of amplification on Vero cells. Master stocks were obtained from Brandy Russell (CDC, Ft. Collins, CO). ZIKV strain Paraiba_01 (ZIKV-BRA; GenBank KX280026) was originally isolated from human serum in Brazil in 2015 with two rounds of amplification on Vero cells, and a master stock was obtained from Kevin Noguchi at Washington University in St. Louis (St. Louis, MO). Virus challenge stocks were prepared by inoculation onto a confluent monolayer of C6/36 mosquito cells. We deep sequenced our virus stocks to verify the expected origin (see next section for details).

### Deep sequencing.

A vial of all viral stocks used for challenges were each deep sequenced by preparing libraries of fragmented double-stranded cDNA using methods similar to those previously described (20, 21, 64). Briefly, the sample was centrifuged at 5,000 relative centrifugal force (rcf) for 5 min. The supernatant was then filtered through a 0.45-μm filter. Viral RNA (vRNA) was isolated using the QIAamp MinElute virus spin kit (Qiagen, Germantown, MD), omitting carrier RNA. Eluted vRNA was then treated with DNase I. Double-stranded DNA was prepared with the Superscript Double-Stranded cDNA synthesis kit (Invitrogen, Carlsbad, CA) and priming with random hexamers. Agencourt Ampure XP beads (Beckman Coulter, Indianapolis, IN) were used to purify double-stranded DNA. The purified DNA was fragmented with the Nextera XT kit (Illumina, Madison, WI), tagged with Illumina-compatible primers, and then purified with Agencourt Ampure XP beads. Purified libraries were then sequenced with 2 × 300-bp kits on an Illumina MiSeq.

### Sequence analysis.

Viral stock sequences were analyzed using a modified version of the viral-ngs workflow developed by the Broad Institute (http://viral-ngs.readthedocs.io/en/latest/description.html) implemented in DNANexus and using bbmap local alignment in Geneious Pro (Biomatters, Ltd., Auckland, New Zealand). Briefly, using the viral-ngs workflow, host-derived reads that map to a human sequence database and putative PCR duplicates were removed. The remaining reads were loaded into Geneious Pro and mapped to NCBI GenBank Zika (GenBank KX601166) reference sequences using bbmap local alignment. Mapped reads were aligned using Geneious global alignment and the consensus sequence was used for intrasample variant calling. Variants were called that fit the following conditions: have a minimum *P* value of 10e−60, a minimum strand bias of 10e−5 when exceeding 65% bias, and were nonsynonymous. Consensus-level nucleotide substitutions and minor nucleotide variants are reported in Table 4.

### Plaque assay.

Quantification of virus titer in maternal serum, placenta, and fetuses were completed by plaque assay on Vero cells. Duplicate wells were infected with 0.1 ml aliquots from serial 10-fold dilutions in growth medium and virus was adsorbed for 1 h. After incubation, the monolayers were overlaid with 3 ml containing a 1:1 mixture of 1.2% oxoid agar and 2× DMEM (Gibco, Carlsbad, CA) with 10% (vol/vol) FBS and 2% (vol/vol) Antibiotic Antimycotic solution. Cells were incubated at 37°C in 5% CO_2_ for 3 days (ZIKV-PR, ZIKV-BRA, and ZIKV-CAM), 4 days (ZIKV-PAN), or 5 days (ZIKV-MEX) for plaque development. Cell monolayers were then stained with 3 ml of overlay containing a 1:1 mixture of 1.2% oxoid agar with 4% neutral red (Gibco) and 2× DMEM with 2% (vol/vol) FBS, and 2% (vol/vol) Antibiotic Antimycotic solution. Cells were incubated overnight at 37°C in 5% CO_2_ and plaques were counted.

### Mice.

Female *Ifnar1*^−/−^ mice on the C57BL/6 background were bred in the specific pathogen-free animal facilities of the University of Minnesota within the College of Veterinary Medicine. Male C57BL/6 mice were purchased from Jackson Laboratories. Timed matings between female *Ifnar1^−/−^* mice and male C57BL/6 mice resulted in *Ifnar1^+/−^* progeny.

### Subcutaneous inoculation.

All pregnant dams were between 6 and 10 weeks of age and were randomly assigned to infected or control groups. Matings between *Ifnar1^−/−^* dams and wild-type sires were timed by checking for the presence of a vaginal plug, indicating gestational age E0.5. At embryonic day 7.5 (E7.5) dams were inoculated in the right hind footpad with 1 × 10^3^ PFU of the selected ZIKV strain in sterile phosphate-buffered saline (PBS) or with sterile PBS alone to serve as experimental controls. All animals were closely monitored by laboratory staff for adverse reactions and/or clinical signs of disease. A submandibular blood draw was performed at 2, 4, and 7 days postinoculation (dpi), and serum was collected to verify viremia. Mice were humanely euthanized and necropsied at E14.5.

### Mouse necropsy.

Following inoculation with ZIKV or PBS, mice were sacrificed at E14.5. Tissues were carefully dissected using sterile instruments that were changed between each mouse to minimize possible cross contamination. Each organ and neonate was morphologically evaluated *in situ* prior to removal. Using sterile instruments, the uterus was removed and dissected to remove individual concepti. Each conceptus was placed in a sterile culture dish and dissected to separate the fetus and the placenta, when possible, for gross evaluation. Fetuses were characterized as “normal” or “resorbed,” with the latter being defined as having significant growth retardation and reduced physiological structure compared to littermates and controls, accompanied by clearly evident developmental delay or visualization of a macroscopic plaque in the uterus. A subset of fetuses and placentas from each litter were reserved for viral titer analysis (preserved in PBS supplemented with 20% FBS and 1% Antibiotic Antimycotic) or fixed in 10% neutral buffered formalin for imaging and histology.

### Crown-to-rump length.

Crown-to-rump length (CRL) was measured by tracing the distance from the crown of the head to the base of the tail, using ImageJ. Infection-induced resorbed fetuses were excluded from measurement analyses because they would not survive if the pregnancy was allowed to progress to term (20).

### Histology.

Placental tissues were fixed in 10% neutral buffered formalin at room temperature for 36 to 48 h and then transferred to 70% ethanol until alcohol processed and embedded in paraffin. Paraffin sections (5 μm) were stained with hematoxylin and eosin (H&E) and the degree of pathology was scored by a blinded pathologist, as described in reference 20. The degree of placental pathology was rated on a relative scale of 0 to 4, where zero represents normal histologic features and 4 represents the most severe features observed. Each zone of the placenta was scored individually for general overall pathology, amount of inflammation, and amount of vascular injury. Only “general” scores are shown because they were representative of “inflammation” and “vascular injury” scores.

### *In situ* hybridization.

Immediately following necropsy, fetuses were fixed in 10% neutral buffered formalin at room temperature for 36 to 48 h and then transferred to 70% ethanol until alcohol processed and embedded in paraffin. Paraffin sections (5 μm) were deparaffinized and a hydrogen peroxide quench was performed, followed by boiling in target retrieval reagent (catalog 322000). Tissue was then incubated in Protease Plus solution (catalog 322330) in a HybEZ II oven at 40°C before hybridization with the ZIKV probe (catalog 468361) and chromogen labeling using the RNAscope 2.5 HD Red assay (catalog 322360). *In situ* hybridization (ISH) was performed using the RNAscope assay with products and instructions (65) provided by the manufacturer (Advanced Cell Diagnostics. Inc., Newark, CA). Each ISH run included ZIKV-infected positive-control tissue to confirm the protocol was run properly. After labeling, tissue was counterstained using hematoxylin before receiving a cover slip for evaluation.

### Fetal and placental viral titers.

An Omni TH115 homogenizer (Omni International, Omni Tissue Homogenizer (TH), 115V) was used to homogenize fetus and placenta samples following necropsy. Samples were submerged in chilled PBS supplemented with 20% FBS and 1% Antibiotic Antimycotic solution in 15 ml Omni sealed plastic tubes (Omni International, catalog 00-2015-25). Omni soft tissue probes (Omni International, catalog 30750) were used to homogenize samples at the highest speed for 15 s (placentas) or 30 s (fetuses). Homogenized samples were clarified by centrifugation at 10,000 × *g* for 2 min. The supernatant was removed and 0.1 ml was immediately plated for plaque assay. The remainder was stored at −80°C.

### Innate immune gene RT-QPCR in mouse placenta.

RNA was extracted and purified from placentas using a Direct-zol RNA kit (Zymo Research). The High-Capacity RNA-to-cDNA kit (Applied Biosystems) was used to synthesize cDNA. Quantitative PCR (qPCR) using PowerUp SYBR green Master Mix (Applied Biosystems) was used to quantify innate immune genes and run on a QuantStudio 3 (Applied Biosystems). The following PrimeTime Primers (Integrated DNA Technologies) were used: *Ifnλr1*: Mm.PT.58.10781457; *Ifnλ2*: Mm.PT.58.31485549; *Ifnλ3*: Mm.PT.58.8956530; *Ifit1*: Mm.PT.58.32674307; *Mx1*: Mm.PT.58.12101853.g; *Oasl2*: Mm.PT.56a.17167264; and *Gapdh*: Mm.PT.39a.1. Innate immune genes were normalized to *Gapdh* and then the threshold cycle value (2-delta delta *C_T_*) was calculated relative to PBS-inoculated controls.

### Data analysis.

All analyses were performed using GraphPad Prism. Unpaired Student's *t* test was used to determine significant differences in crown-rump lengths. Fisher’s exact test was used to determine differences in rates of normal versus resorbed concepti. One-way ANOVA with Tukey’s multiple comparison test was conducted to compare virus titers in maternal serum, placentas, fetuses, and concepti. Nonparametric Spearman correlation was used to evaluate the relationship between variables.

### Data availability.

Virus stock sequence data have been deposited in the Sequence Read Archive (SRA) with accession codes SRX4510825, SRR14467422, and SRR14467421. All other data supporting the findings of this study are available within the article.
